# The genetic associations of COVID-19 on genitourinary symptoms

**DOI:** 10.3389/fimmu.2023.1216211

**Published:** 2023-06-21

**Authors:** Zhenglin Chang, Lingyue An, Min Lei, Zhenfeng Song, Jian Deng, Ruizheng Tang, Zhangkai J. Cheng, Wenqi Wu, Baoqing Sun

**Affiliations:** ^1^ Department of Laboratory, National Center for Respiratory Medicine, National Clinical Research Center for Respiratory Disease, State Key Laboratory of Respiratory Disease, Guangzhou Institute of Respiratory Health, The First Affiliated Hospital of Guangzhou Medical University, Guangzhou, Guangdong, China; ^2^ Department of Allergy and Clinical Immunology, National Center for Respiratory Medicine, National Clinical Research Center for Respiratory Disease, State Key Laboratory of Respiratory Disease, Guangzhou Institute of Respiratory Health, The First Affiliated Hospital of Guangzhou Medical University, Guangzhou, Guangdong, China; ^3^ Department of Laboratory, Guangzhou Laboratory, Guangzhou, China; ^4^ Department of Urology, Guizhou Provincial People’s Hospital, Guiyang, Guizhou, China; ^5^ Department of Urology, The Second Affiliated Hospital of Guangzhou Medical University, Guangzhou, Guangdong, China; ^6^ Guangdong Key Laboratory of Urology, The First Affiliated Hospital of Guangzhou Medical University, Guangzhou, Guangdong, China; ^7^ Department of General Surgery, Ziyang First People’s Hospital, Ziyang, Sichuan, China

**Keywords:** bladder cancer, COVID-19, calculus of lower urinary tract, mendelian randomization, sexual dysfunction, urinary tract infections

## Abstract

**Background:**

Recently emerged reports indicated that patients with coronavirus disease 2019 (COVID-19) might experience novo genitourinary symptoms after discharge. Nevertheless, the causal associations and underlying mechanisms remain largely unclear.

**Methods:**

Genome-wide association study (GWAS) statistics for COVID-19 and 28 genitourinary symptoms with consistent definitions were collected from the COVID‐19 Host Genetic Initiative, FinnGen, and UK Biobanks. Mendelian randomization (MR) analyses were applied to explore the causal effects of COVID-19 on genitourinary symptoms by selecting single-nucleotide polymorphisms as instrumental variables. Meta-analyses were conducted to evaluate the combined causal effect. Molecular pathways connecting COVID-19 and its associated disorders were evaluated by weighted gene co-expression network analysis (WGCNA) and enrichment analyses to extract insights into the potential mechanisms underlying the connection.

**Results:**

The MR and meta-analyses indicated that COVID-19 was causally associated with increased risk for calculus of the lower urinary tract (LUTC, OR: 1.2984 per doubling in odds of COVID‐19, 95% CI: 1.0752–1.5680, *p* = 0.007) and sexual dysfunction (SD, OR: 1.0931, 95% CI: 1.0292–1.1610, *p* = 0.004). Intriguingly, COVID-19 might exert a slight causal protective effect on the progression of urinary tract infections (UTIs) and bladder cancer (BLCA). These results were robust to sensitivity analyses. Bioinformatic analyses indicated that the inflammatory-immune response module may mediate the links between COVID‐19 and its associated disorders at the molecular level.

**Conclusions:**

In response to post-COVID-19 symptoms, we recommend that COVID-19 patients should strengthen the prevention of LUTC and the monitoring of sexual function. Meanwhile, the positive effects of COVID-19 on UTIs and BLCA should attach equal importance.

## 1 Introduction

COVID-19, a disease caused by severe acute respiratory syndrome coronavirus 2 (SARS-CoV-2), has rapidly spread worldwide since its initial outbreak in December 2019 and was declared a global pandemic by the World Health Organization in March 2020 (1, 2). Despite isolation measures, there has been a steep and continuous increase in the morbidity and mortality of COVID-19, and now affecting up to 767 million cumulative confirmed cases and 6.9 million deaths globally. Moreover, the progression of COVID-19 is extremely complicated and often involves host multi-system interactions, even suffering from multiple novo complications after discharge (3, 4). Notably, post-COVID symptoms may result in specific organ dysfunction such as in the lungs or kidneys, and increases the lethality of COVID-19 (3, 4). The ongoing outbreak of COVID-19 and its intractable comorbidities have already consumed a large number of healthcare resources, thus causing considerable public health burden and economic burden on a global scale (2, 5). Therefore, it is necessary to identify to post-COVID-19 symptoms to reduce its disease burden. In particular, a deeper awareness of the causality and effect sizes of COVID-19 on its complications may contribute to the identification of high-risk patients and the design of preventive or therapeutic interventions.

Entry of SARS‐CoV‐2 into host cells is mainly dependent on the receptor angiotensin‐converting enzyme 2 (ACE2) (4, 6). Although SARS-CoV-2 directly invades the lungs, available clinical evidence indicated that the urinary and genital systems are also important targets for SARS‐CoV‐2 infection, due to the ample expression of ACE2 in the genitourinary system (7–9). Several recently emerged surveys have suggested that patients with COVID-19 might experience multiple genitourinary manifestations, including sexual dysfunction (10–12), urolithiasis (13–15), etc. Nevertheless, these associations remain vulnerable to residual confounding and reverse causality in observational studies (16, 17). Mendelian randomization (MR) is a robust method for inferring the causal nature of the exposure on the outcome by adopting the concept of a randomized control trial (16–18). Compared with conventional observational studies, MR could minimize the influence of residual confounding and reverse causality as alleles are randomly assigned and allelic randomization antedates the onset of disease (17).

In the current study, we first collected 28 genitourinary symptoms with consistent definitions from the UK Biobank and FinnGen consortium. Subsequently, we sought to infer potential causal associations of COVID‐19 on genitourinary symptoms using large‐sample statistical data sets of three COVID‐19 subtypes (COVID-19 infection, hospitalized COVID‐19, and severe COVID‐19). Meta-analyses were conducted to evaluate the combined causal effect of COVID-19 on genitourinary symptoms. Finally, molecular pathways connecting COVID-19 and its associated symptoms were evaluated by bioinformatic analyses to extract novel insights into the potential mechanisms underlying the connection.

## 2 Methods

### 2.1 Study design

**Figure 1:**
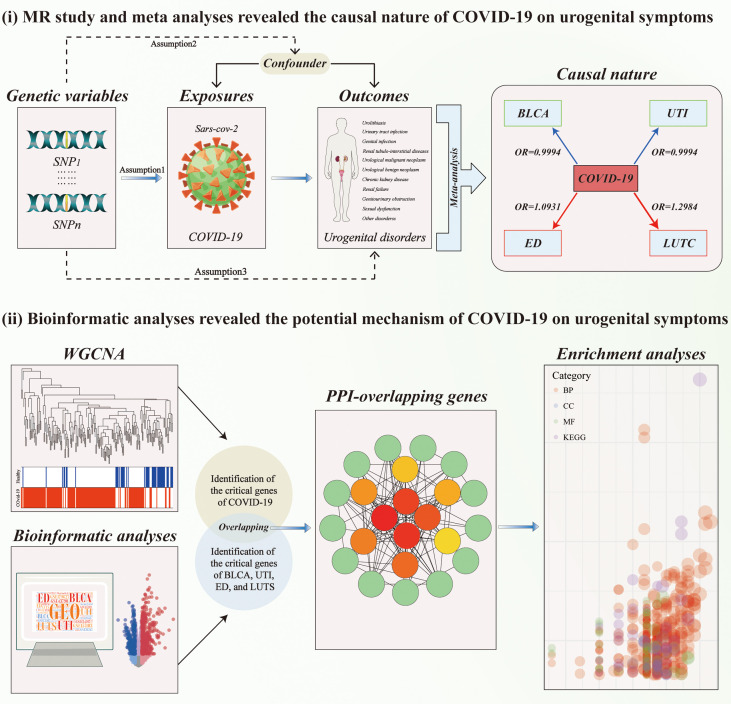
The workflow of designed analysis. MR, Mendelian randomization; BLCA, Bladder cancer; COVID-19, coronavirus disease 2019; LUTC, Calculus of lower urinary tract; SD, Sexual dysfunction; UTI, Urinary tract infection; WGCNA, weighted gene co-expression network analysis.

Two-sample Mendelian randomization (MR) analyses were applied to infer the causal nature of host genetic liability to COVID‐19 on genitourinary symptoms by exploiting single nucleotide polymorphisms (SNPs) as instrumental variables (IVs) of exposure (17, 19). All MR analyses should meet the following three assumptions. First, the selected IVs were robustly associated with the exposure. Second, the used IVs should not be related to potential confounders. Third, the used IVs only affect the outcome via the way of exposure. MR analysis was excluded when pleiotropy was present. There were no additional ethical approval or informed consent was needed as this study was based on the publicly available database. Subsequently, meta-analyses were conducted to evaluate the combined causal effect of COVID-19 on genitourinary symptoms. Finally, molecular pathways connecting COVID-19 infection and its associated disorders were evaluated by bioinformatic analyses to extract novel insights into the potential mechanisms underlying the connection. The workflow of the study design is presented in **Figure 1**.

### 2.2 Genome‐wide association study summary datasets preparation

The exposures were obtained from publicly available GWAS summary results, including those on (COVID-19 infection, 38,984 cases, and 1,644,784 controls), hospitalized COVID‐19 (9,986 cases and 1,887,658 controls), and severe COVID‐19 (5,101 cases and 1,383,342 controls) (20). Genitourinary symptoms were defined as the outcomes. We retrieved the data of genitourinary symptoms from the two nationwide biobanks (UK Biobank and FinnGen) and identified a total of 37 symptoms with consistent definitions after excluding congenital disorders (cystic kidney disease, redundant prepuce, phimosis and paraphimosis, congenital obstructive defects of renal pelvis and congenital malformations of ureter). These disorders were divided into 11 major groups, including urolithiasis, urinary tract infection, genital infection, renal tubulo-interstitial diseases, genitourinary malignant neoplasm, genitourinary benign neoplasm, chronic kidney disease, renal failure, genitourinary obstruction, sexual dysfunction, and other symptoms. Detailed information can be found in **Supplementary Table S3**.

### 2.3 Genetic instrument selection

The candidate IVs were selected by a series of quality control steps. IVs with the genome-wide significance threshold (*P <*5e−8) were picked out and further pruned using a linkage disequilibrium (LD) clumping process (r2 < 0.001, kb > 10,000). Considering that SNPs which directly affect the outcome variable could violate the assumptions of the instrumental variable, any IVs not included in the outcome GWAS and those significantly associated with the outcome (P > 5×10^-5) were removed. Furthermore, the number of above-selected IVs selected from the outcome is not less than three. Subsequently, phenoscanner2 (http://www.phenoscanner.medschl.cam.ac.uk) was used to evaluate whether any exposure-related IVs were associated with confounders of genitourinary symptoms (21). Palindromic and incompatible IVs were then removed by harmonization to ensure that the effect of these IVs on exposure corresponded to the same allele as the effect on the outcome. F statistic was applied to test whether there was a weak instrumental variable bias. The F-statistics were calculated by the formula of F = R^2/(1 − R^2) *(n − k − 1)/k (R^2 = 2*MAF*(1-MAF) *Beta^2; n, sample size; k, number of instrumental variables; and MAF, minor allele frequency). Moreover, the statistical power of MR analysis to detect causal association was calculated by mRnd (22).

### 2.4 Transcriptome datasets preparation

The Gene Expression Omnibus (GEO, www.ncbi.nlm.nih.gov/geo) is a freely available public database containing gene expression for multiple diseases. The GSE157103 dataset collected 126 plasma and leukocyte samples from hospitalized patients with or without COVID-19 (n=100 and 26 respectively) (23). The GSE179627 dataset is peripheral blood mononuclear cell (PBMC) transcriptome data of COVID-19 which contains 22 healthy individuals and 48 COVID-19 patients (24). The GSE13507 dataset contains 188 bladder cancer (BLCA) tissues and 68 non-BLCA controls (25). The GSE43790 dataset contains 5 samples infected by *Escherichia coli* and 3 normal samples (26). The GSE124917 dataset contains 3 samples infected by *Escherichia coli* and 3 normal samples (27). The GSE2457 dataset contains 5 normal and 5 diabetic-ED rats (28). The “ComBat” function of the “sva” package was used to remove batch effects.

### 2.5 Identification of the critical genes of COVID-19

The weighted gene co-expression network analysis (WGCNA) is widely used for finding the co-expressed gene modules with high biological significance and exploring the relationship between gene clusters and diseases (29). Therefore, the “WGCNA” R package was applied to identify the COVID-19-associated modules and genes. First, the hierarchical clustering analysis was conducted using the function of “hcluster” to eliminate outliers. After that, appropriate soft power (β =10) was selected using the “pickSoftThreshold” function according to the standard of a scale-free network. Subsequently, the “adjacency” function was applied to convert to a topological overlap matrix. Hierarchical clustering was then performed using the dynamic shear tree method, resulting in an overall clustering tree of COVID-19 differential genes. By iteratively clustering the eigenvector genes of different modules, modules with high similarity can be obtained, thus constructing a weighted co-expression network of differentially expressed genes. Correlations between gene modules and COVID-19 phenotypes were calculated. Modules with p-values less than 0.01 were regarded as critical gene modules associated with the phenotype and included in the subsequent analysis.

#### 2.5.1 Identification of the critical genes of COVID-19-related disorders

To obtain the critical genes of COVID-19-related disorders, differential analyses were first applied to obtain the differentially expressed gene (DEGs) of BLCA, UTI, and SD. “−log10 (P-value) >1.3 and Log2 (Fold Change) >1 or Log2(Fold Change)<−1” were defined as the threshold for the DEGs. Since only the data on upper urinary tract stones were recorded in the GEO database, we failed to obtain the data on lower urinary tract stones (LUTS). As an alternative, we then searched for LUTS-specific target genes using the GeneCards database (https://www.genecards.org/), the DrugBank database (https://go.drugbank.com/), the PharmGkb database (https://www.PharmGkb.org/), the online Mendelian inheritance in man database (OMIM, http://omim.org/), and the therapeutic target database (TTD, http://db.idrblab.net/ttd/).

#### 2.5.2 Investigating the shared molecular pathways connecting COVID-19 and its associated disorders

To extract insights into the potential mechanisms underlying these associations, we first obtain the overlapping genes between the critical genes of COVID-19 and its associated disorders. To further evaluate the interactions of the overlapping genes, these targets were then put into the STRING database (https://string-db.org/) to construct a protein-protein interaction (PPI) network. Here, we limited lowest interaction score to a medium confidence (0.400), and the species was set to “Homo sapiens”. We eliminated the disconnected targets and then maintained the remaining parameters at default settings. The topological analysis plug-in cytoHubba in Cytoscape software was then applied to display the topological importance of genes. Functional enrichment analyses were applied to annotate the overlapping genes concerning biological process, molecular function, cellular component, and the Kyoto Encyclopedia of Genes and Genomes (KEGG) pathway. The threshold for enrichment analysis was a p-value < 0.05. We excluded terms that were unrelated to our study, including “Hepatitis C”, “Malaria”, “Toxoplasmosis”, etc. Results from enrichment analyses were visualized by the ”GOplot” R package.

### 2.6 Statistical analyses

The “TwoSampleMR” R package based on R (Version 4.0.2) was used to conduct MR analysis. The conventional inverse-variance weighted (IVW) was deemed the most reliable model because it provides the most persuasive estimates when there is no evidence of directional pleiotropy (30–32). Moreover, MR-Egger and weighted-median methods were implemented as sensitivity analysis approaches to ensure the robustness of the results (31, 32). We finally performed the meta-analyses of the MR effect estimates from each of the three cohorts. A random effects model was used where heterogeneity is high (when I2 values were more than 50%); otherwise, a fixed effects model will be used (33). The MR-Egger test for directional pleiotropy and Cochran’s Q statistics were applied to identify whether significant heterogeneity or directional pleiotropy was present. The “limma” R package was used for differential analysis, whereas the “cluster profile” R package was adopted for functional enrichment analysis.

## 3 Results

### 3.1 Information on exposures and outcomes in two nationwide biobanks

Three subtypes of COVID-19 (COVID-19 infection, hospitalization, and severity) were selected as exposures to represent patients infected with SARS-CoV-2. Seven, five, and eight genetic instruments of COVID‐19 infection, hospitalization, and severity were identified after LD pruning, and all of these exposures had strong genetic instruments (F statistics>10, **Supplementary Tables 1**, **2**). Subsequently, we obtained 28 genitourinary symptoms with consistent definitions from two nationwide biobanks (**Supplementary Table 3**).

### 3.2 The MR and meta-analyses revealed the causal roles of COVID-19 on genitourinary symptoms

**Figure 2:**
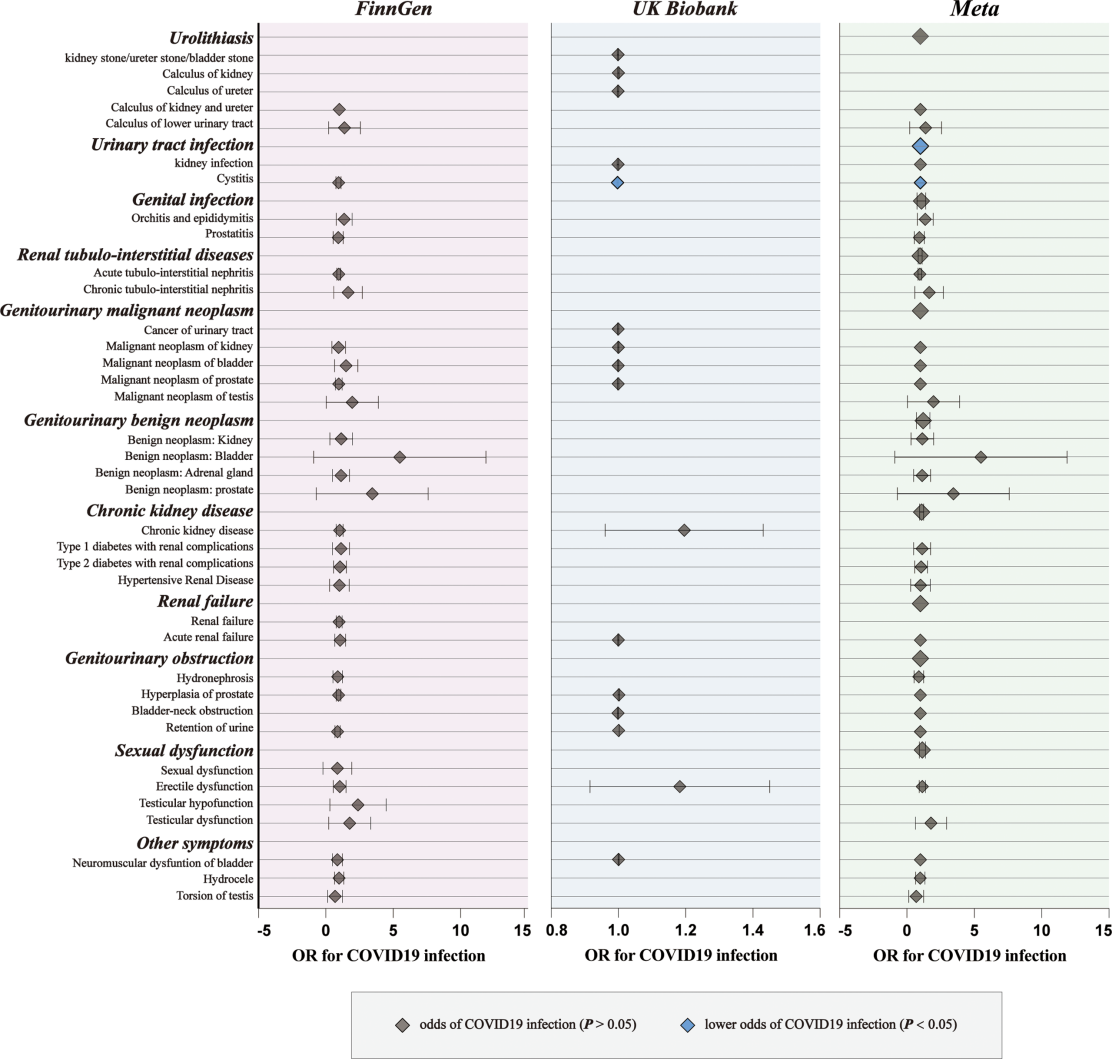
The causal association of COVID-19 infection on genitourinary symptoms. Forest plot for causal effects of COVID-19 infection on genitourinary symptoms in FinnGen and UKB cohort. The meta-analysis of the association results from the two cohorts by the inverse-variance weighted method. The diamonds refer to the point estimates, and the horizontal bars represent the 95% confidence interval. The effect on the x-axis is the odds ratio of genitourinary disorder per 1 standard deviation change in the exposure. The blue diamonds indicated lower odds of genitourinary symptoms (*P* < 0.05) and the grey diamonds indicated the odds of genitourinary symptoms (*P* > 0.05).

**Figure 3:**
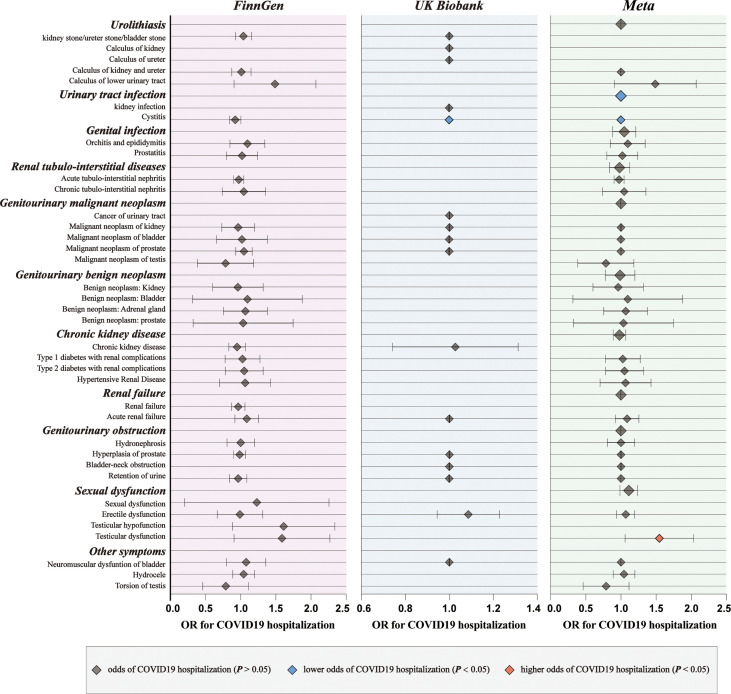
The causal association of hospitalized COVID-19 on genitourinary symptoms. Forest plot for causal effects of hospitalized COVID-19 on genitourinary symptoms in FinnGen and UKB cohort. The meta-analysis of the association results from the two cohorts by the inverse-variance weighted method. The red diamonds indicated higher odds of genitourinary symptoms (*P* < 0.05).

**Figure 4:**
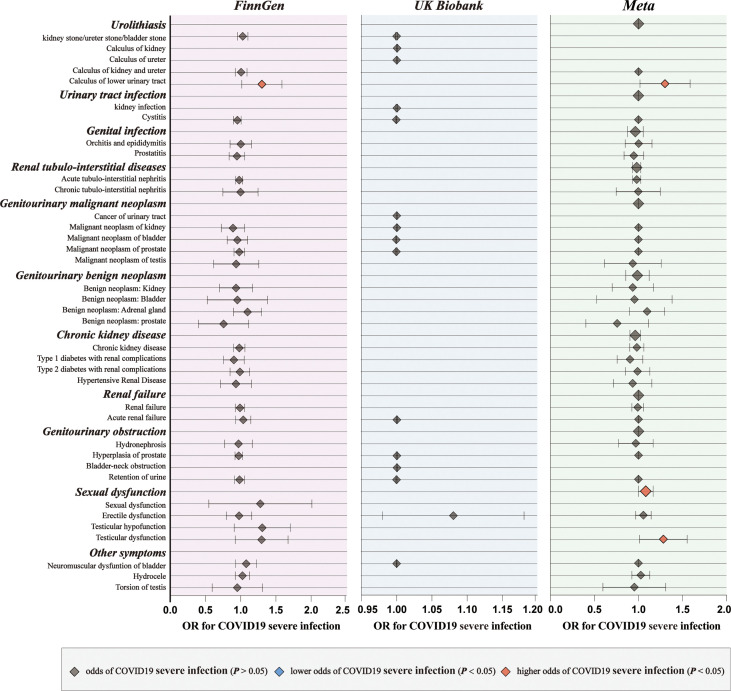
The causal association of serve COVID-19 on genitourinary symptoms. Forest plot for causal effects of serve COVID-19 on genitourinary symptoms in FinnGen and UKB cohort. The meta-analysis of the association results from the two cohorts by the inverse-variance weighted method.

For genetic liability to COVID-19 infection, significant associations with reduced urinary tract infection (OR: 0.9981 per doubling in odds of COVID-19 infection, 95% CI: 0.9968–0.9994, *p* = 0.004) were found after combing the MR results in FinnGen and UKB cohorts (**Figure 2**; **Supplementary Tables 4**, **5**). Genetic liability to hospitalized COVID-19 was significantly associated with reduced urinary tract infection (OR: 0.9992 per doubling in odds of hospitalized COVID-19, 95% CI: 0.9984–1.0000, *p* = 0.04) and increased testicular dysfunction (OR: 1.4954, 95% CI: 1.0866–2.0579, *p* = 0.01) after combing the MR results in FinnGen and UKB cohorts (**Figure 3**; **Supplementary Tables 6**, **7**). For genetic liability to serve COVID-19, significant associations with increased calculus of lower urinary tract (OR: 1.2807 per doubling in odds of served COVID‐19, 95% CI: 1.0280–1.5955, *p* = 2.74e-02) and increased sexual dysfunction (SD, OR: 1.0808, 95% CI: 1.0010–1.1670, *p* < 0.05) after combing the MR results in FinnGen and UKB cohorts (**Figure 4**; **Supplementary Tables 8**, **9**). Heterogeneity was observed in some results with a Cochran Q-derived P value < 0.05 (**Supplementary Tables 4**, **6**, **8**). Given that we used the random-effects IVW as the primary result, heterogeneity is acceptable (34, 35). Moreover, for genetic liability to COVID-19 infection, hospitalization, and severity, no significant associations with other genitourinary symptoms were found after combing the MR results in FinnGen and UKB cohorts (**Figures 2** **-** **4**, **5B**).

**Figure 5:**
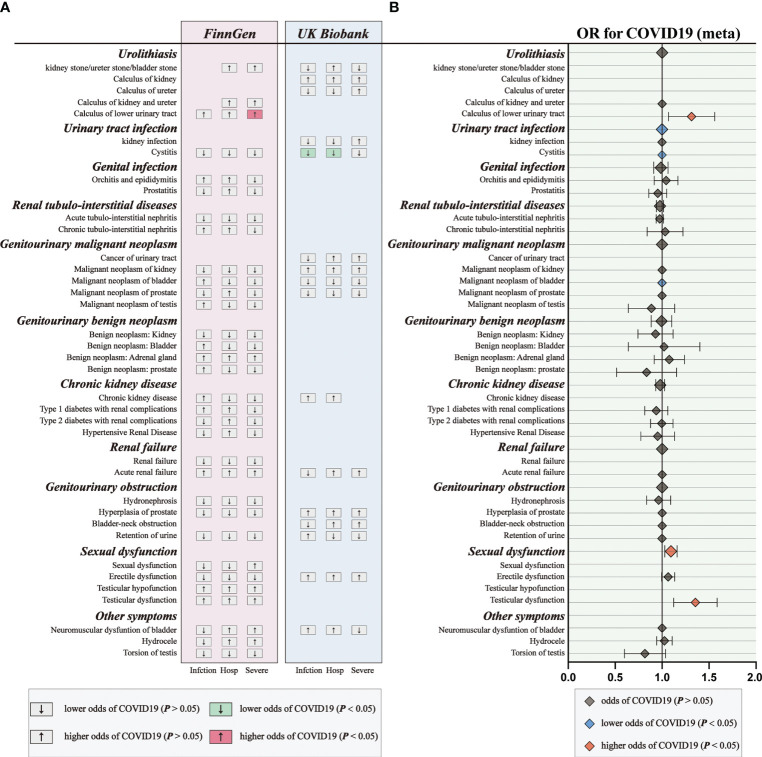
The causal association of COVID-19 on genitourinary symptoms. **(A)** Overview of the associations of COVID-19 on genitourinary symptoms. **(B)** The meta-analysis of the association results from the two cohorts by the inverse-variance weighted method.

Subsequently, we performed meta-analyses to investigate whether these associations are shared across all patients with COVID‐19. We observed that genetic liability to COVID‐19 was significantly associated with reduced risk for urinary tract infection (OR: 0.9994, 95% CI: 0.9989–0.9998, *p* = 0.005), reduced risk for bladder cancer (OR: 0.9994, 95% CI: 0.9989–0.9999, *p* = 0.02), increased risk for calculus of lower urinary tract (OR: 1.2984, 95% CI: 1.0752–1.5680, *p* = 0.007) and increased risk for sexual dysfunction (OR: 1.0931, 95% CI: 1.0292–1.1610, *p* = 0.004) after combing the MR results of three subtypes of COVID-19 (**Figure 5**; **Supplementary Tables 10**).

### 3.3 WGCNA identified the critical genes of COVID-19

**Figure 6:**
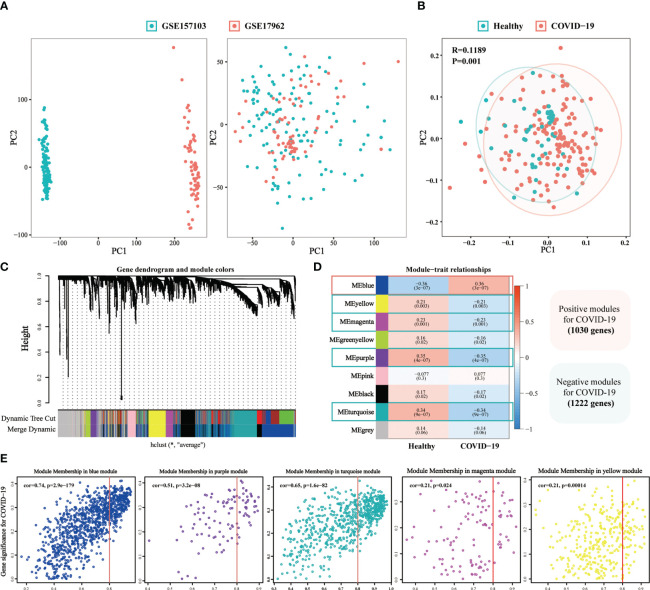
The weighted gene co-expression network (WGCNA) identifies the critical genes of COVID-19. **(A)** The Scatter plots of before and after data standardization. **(B)** Principal component analysis of two GEO datasets. **(C)** Similar gene modules were merged in WGCNA by the dynamic shearing tree method. **(D)** Heatmap revealing module features associated with COVID-19. **(E)** Correlation dot plots of gene significance (GS) value and module membership (MM) value for the selected critical modules in COVID-19.

A total of 148 COVID-19 samples and 48 non-COVID-19 samples were collected from two GEO datasets (**Supplementary Table 11**). Scatter plots indicated that batch effects from different datasets were eliminated (**Figure 6A**). Moreover, we observed the significant differences between the COVID-19 and non-COVID-19 samples (*p* = 0.001, **Figure 6B**). To identify the critical genes of COVID-19, the gene expression profile of complex biological processes was divided into several highly correlated signature modules by WGCNA analysis. The dynamic shearing tree method was then performed to identify and merge the similar gene modules and a total of 9 modules were identified (**Figure 6C**). The purple module (R =0.35, *P* = 4e-07), the turquoise module (R =0.34, *P* = 9e-07), the magenta module (R =0.23, *P* = 0.001), and the yellow module (R =0.21, *P* = 0.003) was negative correlated with COVID-19 while the blue module (R =0.36, *P* = 3e-07) was positive correlated with COVID-19 (**Figures 6D, E** **)**.

#### 3.3.1 Investigating the shared molecular pathways connecting COVID-19 and BLCA

**Figure 7:**
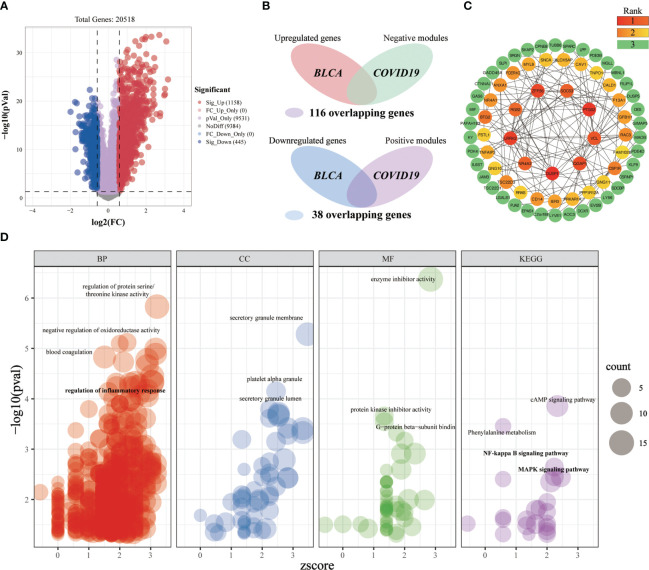
Investigating the shared molecular pathways connecting COVID-19 and BLCA. **(A)** The volcano plot reveals the differentially expressed genes. **(B)** Venn diagram of the crossover genes. **(C)** The topological importance of the overlapping genes was calculated by the topological analysis plug-in cytoHubba. Various colors represent various topological importance of the genes. **(D)** Significantly enriched terms for crossover genes. Res circles refer to the biological process, blue circles indicate the cellular component, green circles mean the molecular function and purple circles denote the pathway. The circle size implies the number of genes enriched in each term.

Differential expression analysis was first conducted in the BLCA-microarray data, which yielded 1158 significantly upregulated genes and 445 significantly downregulated genes (**Figure 7A**). After intersecting the critical targets of BLCA and COVID-19, we constructed two Venn diagrams having the intersected targets and a total of 154 crossover genes (**Figure 7B**). Based on the target screening data, we established a PPI network o have a better understanding of the sophisticated functional networks between the crossover genes (**Supplementary Figure 1A**). The topological importance of the overlapping genes was then displayed by using the topological analysis plug-in cytoHubba in Cytoscape software (**Figure 7C**). To identify the differentially affected functions by the crossover genes, we performed the enrichment analysis through R (**Figure 7D**). After excluding unrelated terms, the overlapping genes were predicted to be related to 730 terms, mainly involved in the regulation of protein serine/threonine kinase activity, negative regulation of oxidoreductase activity, blood coagulation, regulation of inflammatory response, platelet alpha granule, cAMP signaling pathway, NF-kappa B signaling pathway, MAPK signaling pathway, etc. We can roughly divide these terms into modules of inflammatory-immune responses, coagulation-related events, oxidative stress, and other events, which might play a non-neglected role in the association of COVID-19 on BLCA.

#### 3.3.2 Investigating the shared molecular pathways connecting COVID-19 and UTI

**Figure 8:**
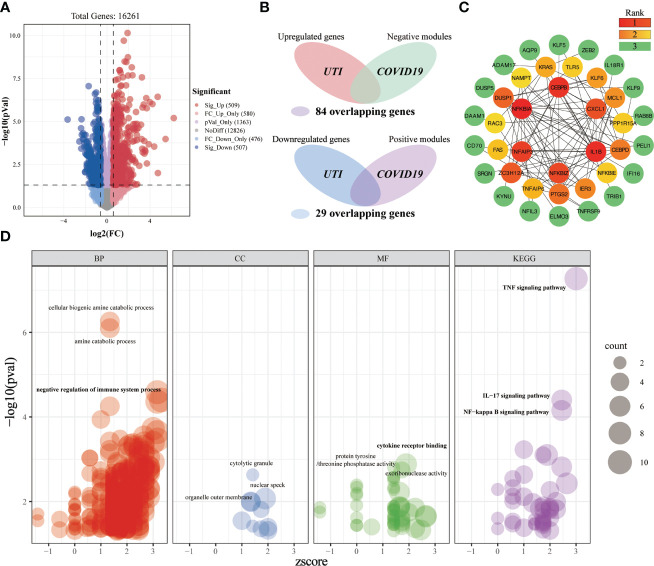
Investigating the shared molecular pathways connecting COVID-19 and UTI. **(A)** The volcano plot reveals the differentially expressed genes. **(B)** Venn diagram of the crossover genes. **(C)** The topological importance of the overlapping genes **(D)** Significantly enriched terms for crossover genes.

A total of 509 significantly upregulated genes and 507 significantly downregulated genes were identified in the UTI-microarray data (**Figure 8A**). After intersecting the critical targets of BLCA and COVID-19, we yielded 113 overlapping genes (**Figure 8B**). Subsequently, we established a PPI network of the overlapping targets (**Supplementary Figure 1B**), and the importance of genes was displayed (**Figure 8C**). In the enrichment analysis, a total of 513 terms were obtained based on the screening criteria (**Figure 8D**). These crossover genes are predominantly enriched in the cellular biogenic amine catabolic process, amine catabolic process, negative regulation of immune system process, cytokine receptor binding, TNF signaling pathway, IL-17 signaling pathway, NF-kappa B signaling pathway, etc. We can roughly divide these terms into modules of inflammatory-immune responses, catabolic process, and other events, which might play a non-neglected role in the association of COVID-19 on UTI.

#### 3.3.3 Investigating the shared molecular pathways connecting COVID-19 and ED

**Figure 9:**
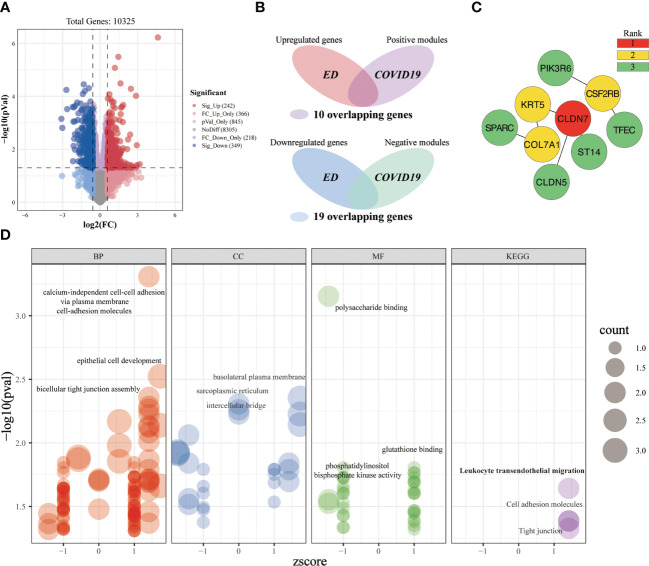
Investigating the shared molecular pathways connecting COVID-19 and ED. **(A)** The volcano plot reveals the differentially expressed genes. **(B)** Venn diagram of the crossover genes. **(C)** The topological importance of the overlapping genes **(D)** Significantly enriched terms for crossover genes.

A total of 242 significantly upregulated genes and 349 significantly downregulated genes were obtained from the ED-microarray data (**Figure 9A**). After intersecting the critical genes of ED and COVID-19, we yielded 29 overlapping genes (**Figure 9B**). Subsequently, we established a PPI network of the overlapping targets (**Supplementary Figure 1C**), and the importance of genes was displayed (**Figure 9C**). Gene enrichment analysis of key genes identified 169 terms (**Figure 9D**). These overlapping genes are predominantly involved in calcium-independent cell-cell adhesion via plasma membrane cell-adhesion molecules, epithelial cell development, bicellular tight junction assembly, polysaccharide binding, leukocyte transendothelial migration, cell adhesion molecules, tight junction, etc. We can roughly divide these terms into modules of cell adhesion, tight junction, inflammatory-immune responses, and other events, which might play a non-neglected role in the association of COVID-19 on ED.

#### 3.3.4 Investigating the shared molecular pathways connecting COVID-19 and LUTC

**Figure 10:**
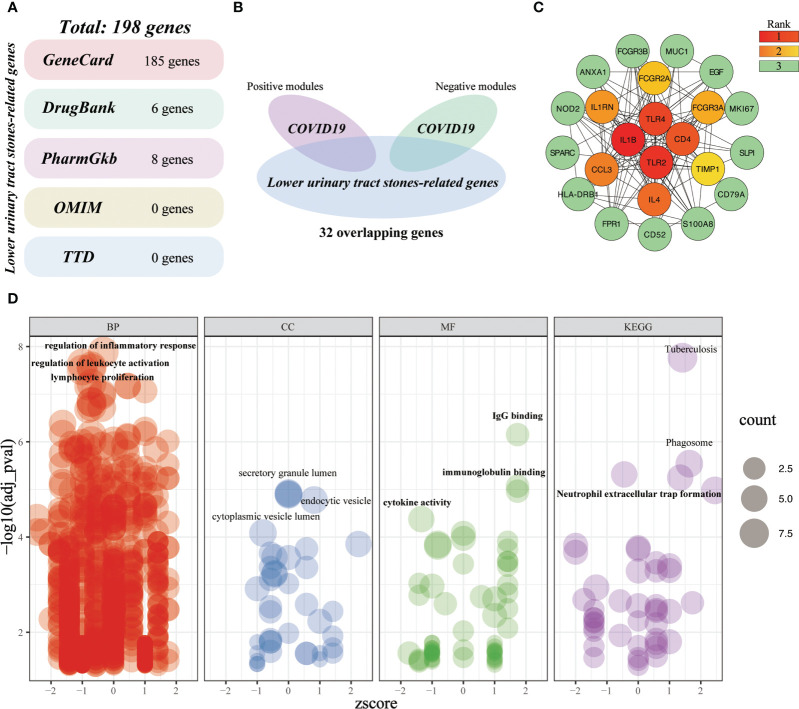
Investigating the shared molecular pathways connecting COVID-19 and LUTC. **(A)** Identification of LUTC-specific target genes from five databases. **(B)** Venn diagram of the crossover genes. **(C)** The topological importance of the overlapping genes **(D)** Significantly enriched terms for crossover genes.

A total of 198 LUTC-specific target genes were obtained from five databases (**Figure 10A**). After intersecting the critical genes of LUTC and COVID-19, we yielded 32 overlapping genes (**Figure 10B**). Subsequently, we established a PPI network of the crossover targets (**Supplementary Figure 1D**), and the importance of genes was displayed (**Figure 10C**). Gene enrichment analysis of overlapping genes identified 1021 terms (**Figure 10D**). These overlapping genes are mainly related to the regulation of inflammatory response, positive regulation of leukocyte activation, lymphocyte proliferation, IgG binding, immunoglobulin binding, cytokine activity, phagosome, neutrophil extracellular trap formation, etc. These results indicated that inflammatory-immune response is a key bridge linking LUTS and COVID-19.

## 4 Discussion

COVID-19 is a complicated multi-system syndrome caused by SARS-CoV-2, which not only results in acute respiratory symptoms but also induces histopathological or functional changes in other organs (3, 12). SARS-CoV-2 could bind to ACE2, a critical mechanism through which SARS-CoV-2 invades host organs and triggers infection (4, 6). The SARS-CoV-2 and its new variants are constantly evolving, thus causing multiple intractable clinical manifestations (36). In recent years, increasing numbers of genitourinary symptoms have been reported after SARS‐CoV‐2 infection (10–15). As ACE2 is highly expressed in the genitourinary system (7–9), the genitourinary system has been gradually considered as one of the key targets for SARS‐CoV‐2 infection. Consequently, we should attach enough importance to the impact of COVID-19 on genitourinary symptoms. According to our knowledge, our research is the first comprehensive inference of the causal associations and potential mechanisms of COVID-19 on genitourinary complications in the context of their shared genetics. Our findings underpin the causal roles of COVID-19 on four genitourinary symptoms, and these associations might be partially mediated by inflammatory-immune responses.

In this study, we detected positive genetic correlations between COVID-19 with calculus of the lower urinary tract and sexual dysfunction. A considerable number of surveys have suggested that the risk of urolithiasis might increase in the era of the COVID-19 pandemic (13–15). One of the studies pointed out the higher white blood cell counts in hospitalized patients due to urolithiasis during the COVID-19 pandemic as compared to the urolithiasis patients’ medical records in the same period a year before COVID-19 (14). Although urolithiasis generally is an uneventful disease, it might be fatal if infection accompanies it (15). In response to SARS-CoV-2 infection, the host immune system fights the virus through the activation of immune cells and the release of inflammatory factors, thus leading to the activated host immune state (37). Moreover, the involvement of inflammatory and immune responses in the pathogenesis of urolithiasis has been well documented (38). These researches indicated that the inflammatory and immune responses may be a nonnegligible bridge connecting COVID-19 and urolithiasis, which are further corroborated by our subsequent bioinformatic analyses. Nevertheless, our results indicated that genetic liability to COVID-19 did not confer a causal effect on upper urinary tract stones. The association between COVID-19 and upper urinary tract stones in previous observational studies might be affected by other confounding factors, such as dietary habits and lifestyle changes during the COVID-19 pandemic (14, 39). Another causal effect of COVID-19 on the genitourinary system is increasing the risk of sexual dysfunction, which has attracted the wide attention of scholars at home and abroad in recent years (10–12). The viral particles can be detected in the male reproductive system even up to 7 months following SARS-CoV-2 infection (40). The direct invasion of SARS-CoV-2 usually causes inflammatory responses in the reproductive system (10). Nevertheless, our results suggested that there is no causal relationship between COVID-19 and genital infection, we, therefore, speculated that SARS-CoV-2 might cause subclinical genital inflammatory responses. The concept was supported by other studies, which indicated that low-grade subclinical inflammation has been deemed as the key pathophysiologic mechanism linking erectile dysfunction (ED) and its related diseases (41, 42). Notably, there was a dramatic reduction in urology visits due to fear of SARS-CoV-2 infection (43, 44). Nevertheless, the calculus of the lower urinary tract and sexual dysfunction can bring a considerable burden to patients and their families. In response to post-COVID-19 symptoms, we, therefore, recommend that patients infected with SARS-CoV-2 should strengthen the prevention of calculus of the lower urinary tract and the monitoring of sexual function.

Intriguingly, our results suggested that COVID-19 might exert a slight causal protective effect on the progression of urinary tract infections (UTIs) and bladder cancer (BLCA). Nevertheless, several single-center studies addressing this association of COVID-19 on UTIs have shown contradictory conclusions (45–48). Among these studies, a portion of them indicated that the incidence of UTIs was higher in the year following the onset of the COVID-19 pandemic compared with prior years (48). Another portion observed a low prevalence of UTIs during the COVID-19 pandemic (45–47). Similarly, a recent multicenter cross-sectional study from a large, geographically diverse sample observed a low prevalence of UTIs among febrile patients infected with SARS-CoV-2 (49), which aligned with our finding. In addition, evidence indicated that the incidence of BLCA during the COVID-19 pandemic was lower as compared with other urinary malignancies (50). Some scholars speculated that the moderate expression of ACE2 in the bladder might be related to the low incidence of BLCA observed in previous study (51). In our current study, we provide direct evidence establishing a causal relationship between COVID-19 and a decreased risk of UTI and BLCA. Moreover, we propose that inflammatory and immune responses could serve as potential mediators for these observed associations. This assertion is further supported by our findings that the genes overlapping between COVID-19, UTI, and BLCA are predominantly involved in inflammatory and immune response pathways. Currently, the inflammatory and immune responses have been widely accepted as a double-edged sword, which can not only protect the body but also hurt it. The dysregulated inflammatory responses are associated with immune suppression, usually resulting in disease progression (52–54). Conversely, the normally controlled inflammation may initiate favorable immune responses, thus protecting the organism (52, 55). During urinary tract infection, rapid releases of inflammatory factors are conducive to pathogen clearance, while the excessively released cytokines might aggravate the inflammatory process and tissue damage (53, 54). In the progression of bladder cancer, the normally controlled inflammation could prevent the growth and invasion of the malignancy, while the uncontrolled inflammation might aid the metastatic spread of the tumor (52, 56). Therefore, we speculated that SARS-CoV-2 infection might activate the normally controlled inflammatory and immune responses in the urinary tract, thereby preventing the progression of UTIs and BLCA. The mechanism underlying these protective effects warrants further investigation from genetics and clinical evidence.

Our findings underpin the causal roles of COVID-19 on four genitourinary symptoms, and these associations might be partially mediated by inflammatory-immune responses. In response to post-COVID-19 symptoms, COVID-19 patients should strengthen the prevention of LUTC and the monitoring of sexual function. Meanwhile, the urologist should attach equal importance to the positive effects of COVID-19 on UTI and BLCA, and further research is warranted to decipher whether it is an unknown mechanism, are biologically important in favorable inflammatory-immune responses. However, further biological experiments are needed to confirm these findings and to explore the potential mechanisms underlying these associations.

## Data availability statement

The datasets presented in this study can be found in online repositories. The names of the repository or repositories and accession number(s) can be found in the article or **Supplementary Material**.

## Ethics statement

Ethical review and approval was not required for the study on animals in accordance with the local legislation and institutional requirements. Written informed consent was not required as per local legislation and institutional requirements.

## Author contributions

ZC: Conceptualization, Investigation, Visualization, Writing - Original Draft. LA: Formal analysis, Writing - Review & Editing. ML: Writing - Original Draft, Formal analysis. ZS: Visualization, Formal analysis. JD: Visualization. RT: Visualization. YZ: Formal analysis. ZJC: Writing - Review & Editing. WW: Funding acquisition. BS: Project administration, supervision. All authors contributed to the article and approved the submitted version.

## Supplementary material

Supplementary Figure 1 The PPI network of crossover genes.
