# Supplementary figures and images for: The genetic associations of COVID-19 on genitourinary symptoms
Source: Front Immunol. 2023 Jun 21;14:1216211. doi: 10.3389/fimmu.2023.1216211 (PMC10319997; doi:10.3389/fimmu.2023.1216211)

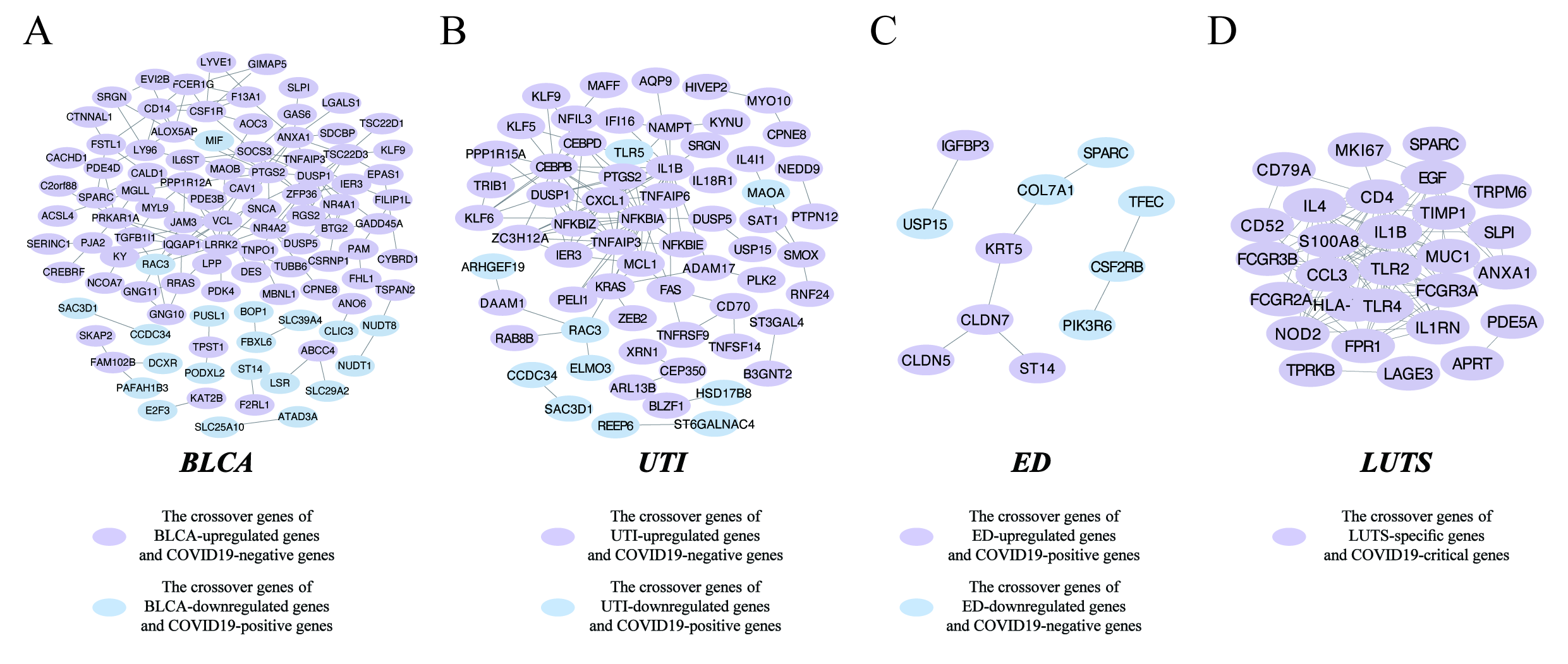

Supplement: Supplementary Figure 1 — The PPI network of crossover genes. [file DataSheet_1.zip › Supporting Information/ChangFigS1.tif]
