# Supplementary material for: The genetic associations of COVID-19 on genitourinary symptoms
Source: Front Immunol. 2023 Jun 21;14:1216211. doi: 10.3389/fimmu.2023.1216211 (PMC10319997; doi:10.3389/fimmu.2023.1216211)
Supplement: Supplementary file 2 [file DataSheet_2.docx]

https://www.jianguoyun.com/p/DbskFz8Q_NKICBihzZcEIAA
